# Metabolomics of Breast Cancer: A Review

**DOI:** 10.3390/metabo12070643

**Published:** 2022-07-13

**Authors:** Ramadevi Subramani, Seeta Poudel, Kenneth D. Smith, Adriana Estrada, Rajkumar Lakshmanaswamy

**Affiliations:** 1 Center of Emphasis in Cancer, Department of Molecular and Translation Medicine, Paul L. Foster School of Medicine, Texas Tech Health Science Center El Paso, El Paso, TX 79905, USA; ramadevi.subramani@ttuhsc.edu (R.S.); sepoudel@ttuhsc.edu (S.P.); adriana.estrada@ttuhsc.edu (A.E.); 2 L. Frederick Francis Graduate School of Biomedical Sciences, Texas Tech Health Science Center El Paso, El Paso, TX 79905, USA; kenneth.d.smith@ttuhsc.edu

**Keywords:** breast cancer, metabolomics, metabolites, glycolysis

## Abstract

Breast cancer is the most commonly diagnosed cancer in women worldwide. Major advances have been made towards breast cancer prevention and treatment. Unfortunately, the incidence of breast cancer is still increasing globally. Metabolomics is the field of science which studies all the metabolites in a cell, tissue, system, or organism. Metabolomics can provide information on dynamic changes occurring during cancer development and progression. The metabolites identified using cutting-edge metabolomics techniques will result in the identification of biomarkers for the early detection, diagnosis, and treatment of cancers. This review briefly introduces the metabolic changes in cancer with particular focus on breast cancer.

## 1. Introduction

Breast cancer is the most common cancer in women and it is one of the leading causes of cancer-related death among women. There is already strong evidence that multiple risk factors make women more susceptible to breast cancer. Even so, the incidence of breast cancer is increasing in women globally [1,2]. Reproductive factors such as early menarche, late menopause, and age at first full-term pregnancy influence the risk of breast cancer [3]. Beside these, other factors such as obesity or first-degree family history of breast cancer also alter the risk of breast cancer [4]. Family history of breast cancer increases the chances of getting breast cancer by 2.5-fold or higher [5,6]. Having the BRCA1 and BRCA2 mutation also puts women at an even higher risk [7]. Some other known risk factors include body fat, alcohol consumption, and high child birth weight [3,8]. There is a 40% increase in the risk of breast cancer in obese postmenopausal women [9]. There are even non-genetic, modifiable risk factors which affect the risk of breast cancer, such as income, education, and insurance status [10]. Ethnicity is another significant risk factor, with breast cancer predominately affecting Non-Hispanic white females, followed by Non-Hispanic blackfemales [11]. Although the risk of breast cancer is lower in Hispanics, according to the American Cancer Society Cancer Facts and Figures for Hispanics/Latinos 2018–2020, it is still the most frequently diagnosed cancer and is the leading cause of cancer-related death, as well in this group of women. Interestingly, it is also well-known that majority of women who are newly diagnosed with breast cancer do not have any known risk factors [12].

The World Health Organization estimates that breast cancer accounted for 12% of all new cancers diagnosed worldwide in 2021 [2]. By 2030, it is estimated that the number of breast cancer cases worldwide will increase to almost 3.2 million new cases per year [13]. It is also estimated that, in the US, 287,850 new cases of invasive breast cancer and 51,700 new cases of non-invasive breast cancers will be diagnosed in 2022. Approximately 43,250 women are expected to die due to breast cancer in 2022 in the US. The five-year survival rate of breast cancer varies depending on stage and subtype of breast cancer.

## 2. Breast Cancer Subtypes

Breast cancer is a highly complex and heterogeneous disease with very distinctive characteristics. It also has an array of clinical presentations and responses to therapy [14]. They are mainly classified based on histological and molecular subtypes [15,16,17]. Histological subtypes include mainly ductal and lobular carcinomas. Besides ductal and lobular breast carcinomas, other histological types include: mucinous, tubular, comedo, inflammatory, medullary and papillary carcinomas, which account for about 10% of the cases [18]. The molecular classification of breast cancer was made using microarray-based gene expression analysis and unbiased hierarchical clustering. Based on these findings, breast cancers are classified as luminal subtype A (estrogen receptor (ER)-positive and/or progesterone receptor (PR)-positive, human epidermal growth factor receptor 2 (HER2)-negative and low Ki67), luminal subtype B (ER-positive, PR-positive or negative, HER2-positive and has high Ki-67), HER2-positive (HER2 overexpressing), basal-like/triple-negative (ER-negative, PR-negative and HER2-negative), normal breast-like and claudin-low [16,19]. Over 60% of all breast cancers are ER+, 15–20% are HER2+ and 10–20% accounts for triple-negative breast cancer (TNBC) [20,21,22]. Generally, hormone-receptor-positive breast cancers are less aggressive than hormone-receptor-negative breast cancers. Even though breast cancer-related death has decreased by 1.0% from 2013 to 2018 [23], the incidence rate for invasive breast cancer has increased approximately by 0.5% annually in the US [2,24]. There are several reasons that have been attributed to the increased incidence of breast cancer worldwide, which include adoption of a Western lifestyle, delayed childbearing, combined hormone replacement therapy, etc. [25]. Another reason for this increase in breast cancer incidence is that early diagnosis and treatment are still challenging.

## 3. Metabolome and Metabolomics

**Figure 1:**
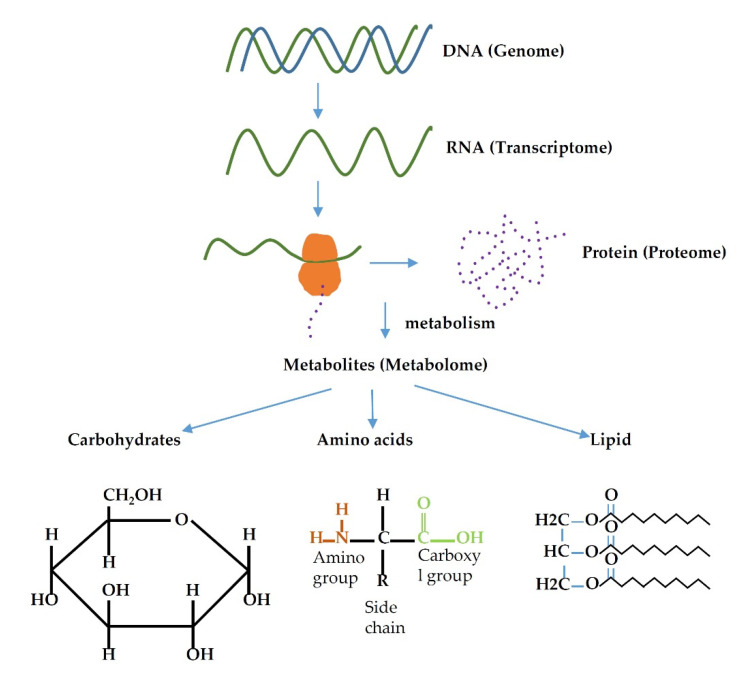
Relation flow of different omics family.

The term metabolome/metabolomics was coined in 1998 [26]. The metabolome is the total number of metabolites present within the cell, tissue, organ, or organism and have a wide range of functions. Metabolomics is a relatively new field focusing on the analysis of metabolites in the metabolome. The message in the DNA (genome) of an organism are transcribed into RNA (transcriptome), translated into protein (proteome) and finally results in the formation of small molecules (result of metabolism) known as metabolites (metabolome) (Figure 1). Thus, any change in the gene, whether that be mutation, over-expression, or under-expression, alters the metabolomics profile of an organism. Many diseases, including cancer, are the result of alteration in gene expression profile, e.g., *BRAC1*/*BRAC2* genes are the most mutated genes in hereditary breast cancer [27]. Therefore, alterations in genes could cause changes in the metabolic profile, and these changes eventually could facilitate cancer development.

**Figure 2:**
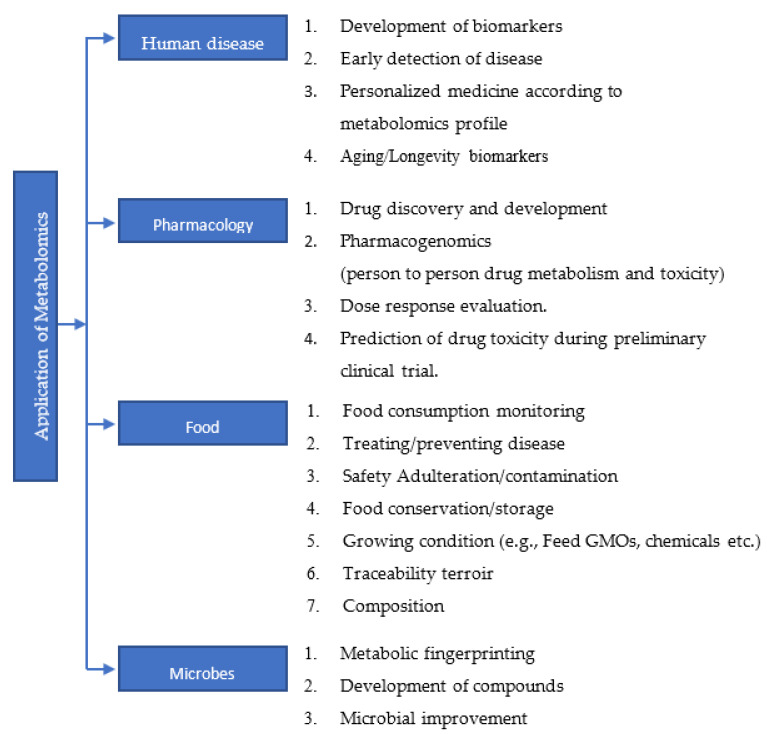
A brief list of applications of metabolomics study in various fields.

The metabolome includes all the products of catabolism and anabolism. Like genomics, transcriptomics, and proteomics, metabolomics study has also gained its own importance. Metabolites are closely linked to the phenotype of an organism; a metabolome is largely defined by its genome. Metabolomics or metabolic profiling is the measurement in biological systems of low-molecular-weight metabolites and intermediates that reflect the dynamic response to genetic modification. It is considered as a very powerful and reliable tool that has high reproducibility, and which can have significant impact on the health of humans [28]. A comprehensive metabolomics study using 928 cell lines from over 20 different cancer types resulted in the identification of 225 metabolites specific to cancer metabolism [29]. Changes in the levels of metabolites can be used as diagnostic markers [30,31], prognostic markers [32] and also as therapeutic targets [33,34,35]. Many biological processes associated with age, gender, obesity, medication, cancers, cardiovascular disease, diabetes, etc., can alter the metabolomics profile of an individual [36,37,38]. The study carried out by Mansell et al. highlighted how the metabolome profile can be used as a marker to predict the future risk of cardiovascular disease in a new born child of a mother with gestational diabetes [39]. Further, epidemiological and experimental data demonstrate that critical changes in metabolites are observed during aging, age-related diseases and geriatric syndromes [40]. It is well-known that various types of cancers are age-related diseases. Endocrine and mitochondrial functions, signaling pathways and calorie restrictions in aging have been associated with amino acid metabolism, lipid metabolism, redox homeostasis and nutrient sensing [41,42,43,44]. Since changes in metabolomics profiles can be associated with various pathological conditions, studying the metabolome will be of significant help towards the development of personalized medicine and also in the early detection and diagnosis of diseases. Application of metabolomics study in various field is summarized in flow chart (Figure 2).

## 4. Analysis of the Metabolome

Metabolites can be measured by 3 major techniques: gas chromatography-mass spectrometry (GC-MS), liquid chromatography (LC-MS), and nuclear magnetic resonance spectroscopy (NMR) [45]. There are two approaches in mass spectrometry: untargeted and targeted. An untargeted approach focuses on a broad range of metabolites; these metabolites are then identified and characterized. A targeted approach focuses on finding the pre-defined and characterized metabolites. In general, the targeted approach has higher sensitivity and selectivity. NMR is a reliable and sensitive technique, which can detect relatively small changes in metabolite concentration among biological specimens [46]. Metabolomics profiling can also be performed and visually observed by using Scanning Electron Microscopy (SEM), Matrix-Assisted Laser Desorption Ionization (MALDI), and Nanostructure-Imaging Mass Spectrometry (NIMS) [47]. The data collected are processed for statistical analysis using various platforms such as XCMS Online, DeviumWEb and MetaboAnalyst [47]. Once metabolites are identified, they are correlated to a particular phenotype, physiological state, or aberration [47]. Metabolites can also be identified by comparing the result obtained from a mass spectral and RI index with databases such as GOLM, NIST05, METLIN, Mass Bank, or the most widely used, Human Metabolome Database (HMDB) [47,48]. As promising as metabolomics sounds, there are a few disadvantages. The latest release by the HMDB contained 247 inborn error metabolites (IEMs) for humans, including both endogenous and exogenous metabolites [49]. There are also the challenges in metabolites extraction, purification, fractionation, and identification. Lastly, there is the possibility of false-positive metabolites identification [50]. Metabolomics is still an emerging field and has a lot of promise to help identify new disease conditions, biomarkers, and therapeutic targets.

## 5. Metabolomics Profile of Breast Cancers

Metabolomics is promising in the area of personalized medicine because it reflects a patient’s phenotype most closely [51]. The cancer metabolome is constituted by different factors. These include metabolites that are the product of the oncologic process, as well as the systemic response of the body to tumors [51]. Studying metabolites and their associated pathways in human diseases such as cancer will provide a better understanding of how the dysregulated metabolism could lead to the initiation and progression of cancer [52]. There are studies that have demonstrated that many types of cancers, including breast cancer, alter the metabolic system in profound ways. In order to understand the development and progression of cancer, it is clearly necessary to understand the cellular metabolome and its metabolic changes [28]. Metabolomics can thus provide a measurement of these phenotypic changes that reflect genetic alterations in breast cancer [52]. An example of this is tumorigenesis, which is a consequence of oncogenic mutations and is dependent on the reprogramming of cellular metabolism. Moreover, since it is well know that cancer cells need to sustain abnormal growth and proliferation rates, which require supplements of metabolic precursors, it makes intuitive sense that it would also lead to an altered metabolism [48]. These secreted metabolites then enter circulation and are transferred to target tissues. These in turn exert biological effects that modulate cells [12]. Metabolic changes play important pathological roles by inducing proliferation, angiogenesis and epithelial-to-mesenchymal transition (EMT). Metabolites can also have a profound effect on mitochondrial metabolism. An example of this is with estrogen, which has been shown to induce carcinogenesis by causing changes in the expression of mitochondrial genes [12]. Metabolome alterations can also be specific to breast cancer subtypes. Metabolomics data has been able to distinguish ER and HER2 molecular subtypes by using glutamate-to-glutamine ratio and aerobic glycolysis as biomarkers [45]. Metabolites can be used as an potential indicator which also describes the aggressiveness of the breast cancer [53]. Similarly, metabolites of the energy-generating metabolic pathways such as glycolysis, TCA cycle, beta-oxidation are higher in hormone-receptor-negative breast cancer and TNBC compared to hormone-receptor-positive breast cancer, which is positively correlated to the aggressiveness of breast cancer [54]. Hence, metabolomics study is important in categorizing breast cancer into different types and stages, as it uses accurate methods and requires less time for analysis. Metabolites from secondary bile acid metabolism, amino acid degradation, short-chain fatty acid production, and deconjugated hormones can be measured to predict tamoxifen resistance, hormone-induced apoptosis, cancer aggressiveness, and histone deacetylase (HDAC) inhibition [55,56,57].

Morphological changes of the cancer cell are also regulated by metabolites such as collagen, cytokines, or by adipocytes in the tumor microenvironment. Currently, more than 30 endogenous metabolites in breast tissue have been identified including elevated choline, low glycerophosphocholine, and low glucose in breast cancer tissue compared to healthy tissue or benign tumors [51]. There are also several pathways that influence the levels of glutamine, lipids, serine, protein translation and cholesterol metabolism, which have been shown to be upregulated in breast cancer [12]. These metabolic changes, and their impact on the progression or inhibition of breast cancer, can serve as a biomarker for early diagnosis and potential target for treatment.

### 5.1. Carbohydrate Metabolism

A hallmark of cancer is the altered utilization of energy by cancer cells relative to normal cells due to increased rates of proliferation [58]. Glycolysis in breast cancer cells is characterized by decreased level of glucose [59,60,61,62] and increased levels of lactate [63,64,65]. Earlier, it had been reported that glucose metabolism was the sole source of energy for cancer cells [66], but more complex processes are required to accomplish this [67]. Cancer cells rely on aerobic glycolysis for energy, popularly known as Warburg effect [68]. This preference for aerobic glycolysis even in the presence of oxygen [68] is an effective way for attaining sufficient energy and evading immune suppression [69]. Furthermore, this could also facilitate complete oxidation of carbon in cancer cells compared to normal cells [59].

The metabolites that are involved in pathways associated with energy metabolism, such as glycolysis, glycogenolysis, tricarboxylic acid cycle (TCA cycle) proliferation and redox pathways are found to be significantly altered in breast cancer [54]. These metabolic processes also produce many metabolites, which are the precursors for many macromolecules required for cancer cell growth and proliferation. Glucose-6-phosphate undergoes the pentose phosphate pathway to yield ribose-5-phosphate precursor for nucleic acid biosynthesis; NADPH and acetyl CoA are used for lipid synthesis; intermediate 3-phosphoglycerate serves as the precursor for synthesis of amino acid such as glycine and cysteine. Targeting the glycolytic enzyme can be beneficial for the inhibiting cancer growth and progression. GLUT-1, a glucose transporter, is present in high levels in TNBC and could serve as potential therapeutic target [70]. Recently, GLUT-1 inhibitor BAY-876 has been shown to selectively inhibit the growth of TNBC cell lines [70]. Aggressive forms of breast cancers tend to have increased rates of glycolysis, TCA cycle, proliferation, and redox pathways [12,71]. It has been well established that metabolites such as ATP, acetyl-coA, and NAD regulate post-translational modifications that affect protein activity adversely, and these co-substrates counteract normal biological pathways. Cancer cells can adapt well to an increase in lactate production and survive in acidic microenvironments [47]. In addition, lower 5-year survival rates have been associated with higher levels of lactate [72]. These findings demonstrate that the metabolites of carbohydrate metabolism play a major role in breast cancer growth and progression. Further understanding the roles and mechanisms by which these metabolites influence breast cancer is of importance.

### 5.2. Lipid Metabolism

Lipid metabolism is significantly altered in cancers. Increased cell growth and tumor formation needs increased synthesis and uptake of lipids. Lipids are key molecules that form the structural basis of biological membranes. They also function as signaling molecules and as an energy source. Several cancers have enhanced fatty acid synthesis, and lipogenesis is vital for tumor growth [73]. Fatty acids are the source of energy as well as the precursor for lipid molecules. The cancer cells undergo two processes: First, de novo synthesis of fatty acid, which utilizes citrate from the TCA cycle and synthesizes many lipid molecules required for cancer cell growth. Second, beta oxidation of exogenous fatty acids, whereby fatty acids are transported into the cell by fatty acid transporters and undergo beta oxidation, yielding huge amounts of energy required for cancer cells. Earlier it was demonstrated that lipid uptake can also be achieved through the receptor-mediated endocytosis of low-density lipoprotein (LDL) particles, CD36 fatty acid translocase, fatty acid transport proteins (FATP) and fatty acid binding proteins (FABP) [74,75,76]. Upregulation of the metabolites that are involved in fatty acid transportation and fatty acid synthesis molecules have been observed in breast cancer [77] and these molecules can be used as biomarkers of breast cancer diagnosis and treatment.

Cancer cells and adipocytes interact to promote tumorigenesis, including breast cancers [78,79]. Coculture of cancer cells with adipocytes increased fatty acid oxidation by activating AMPK activation [80,81,82]. Leptin secreted by adipocytes enhanced fatty acid oxidation by activating JAK/STAT3 signaling pathway in breast cancer stem cells [83]. Acetylcarnitine plays an important role in the production of energy. It transports fatty acid to mitochondria for beta-oxidation and is positively correlated with the risk of breast cancer [84]. Higher levels of acetylcarnitine result in breast cancer development via insulin resistance [84,85]. CD36 is a fatty acid translocase which interacts with FABP4 for transporting fatty acid into the cell. CD36 expression promotes breast cancer by increasing STAT3 signaling and beta oxidation, providing energy for growing breast cancer cell [86]. The inhibition of CD36 and FABP4 induces apoptosis in breast cancer cell lines. Thus CD36 can be used as a biomarker for diagnosis and can also be used as a target for treatment of breast cancer [86]. Phospholipids lysophosphatidlycholine (LPC) correlates with lower risk of breast cancer, while higher level of phosphotidylcholine (PC) correlates with increased risk of breast cancer [87]. Lysophosphatidylcholine acyltransferase 1 (LPCAT1) converts LPC to PC and it is overexpressed in breast cancer with poor prognosis [88], which demonstrates that a higher level of PC is associated with a high risk of breast cancer. Hence, cellular levels of LPC and PC could predict the risk of breast cancer, and targeting LPCAT1 can add a new therapeutic approach for breast cancer. Overall, increased fatty acid synthesis alters cellular lipid composition in cancer cells, and could serve as potential diagnostics and therapeutic targets. The increase in de novo FA synthesis in cancer cells alters cellular lipid composition and can be used for diagnostics [89].

### 5.3. Amino Acid Metabolism

Amino acid metabolism plays a critical role in cancer cell proliferation. Non-essential amino acids and semi-essential amino acids are also needed in addition to essential amino acids to sustain breast cancer cell proliferation [90,91]. It is accomplished by establishing pools of amino acids for the production of non-essential amino acids to be used in protein synthesis, conversion of glucose and lipids, and the activation of key signaling pathways. Amino acids facilitate epigenetic modification, such as methylation, produce α-ketoglutarate, which is oxidized by the tricarboxylic acid (TCA) cycle, oxidative phosphorylation for ATP production, and also maintain intracellular redox status [90]. Amino acid consumption and utilization helps to sustain the growth of cancer cells [92,93,94,95,96].

Glutamine metabolism plays an important role in serving the energy demand of the cancer cell. Multiple transporters, including the Na^+^-dependent transporters, system ASC, which preferentially transports alanine/serine/cysteine and the Na^+^-coupled neutral amino acid transporters (SNATs) import glutamine into cancer cells [97,98]. Tumors have high expression of glutamine transporters ASCT2, SNAT1, SNAT2, and SNAT5 [99]. Inhibition of ASCT2 has been shown to decrease the growth of various cancers including triple-negative breast cancer [100]. Reductive glutamine metabolism for lipid biosynthesis supports tumor growth under hypoxia or mitochondrial dysfunction [101]. In addition, citrate, fumarate and malate derived from glutamine are increased in glucose-deprived cancers, demonstrating that glutamine can support the growth of tumors in a nutrient-poor microenvironment [102]. The glutaminase I (GLS-I) enzyme converts glutamine via glutaminolysis into glutamate, which in turn is converted to alpha ketoglutarate by glutamate dehydrogenase. This alpha ketoglutarate enters the TCA cycle. The glutaminolysis pathway generates a high amount of energy (via TCA cycle) and also produces macromolecules required for cancer cell growth and proliferation. Therefore, the glutamate to glutamine ratio (GGR) can be used as a biomarker for breast cancer diagnosis [103]. A higher GGR has been observed in ER-ve breast cancer and TNBC. Thus GLS-I enzyme can be a target for the treatment of breast cancer. Molecule 7, a derivative of withangulatin A, is an inhibitor of GLS-I enzyme, which inhibited the growth of TNBC cell line by decreasing the amount of glutamate [104].

Serine is a neutral amino acid and is imported into the cells via ASCT1. Breast cancers have high expression of ASCT1 [105]. Serine acts as a carbon source for nucleotide synthesis and as a source for DNA methylation. It has been shown to play a vital role in cancer growth and progression. Increased rates of proliferation in tumor cells relies on the availability of extracellular serine. The reduction in serine and glycine inhibits tumor growth and increases survival time of mice with tumors [106,107]. Some cancers prefer serine as a source of nutrition for rapid cell proliferation while others prefer glycine [93,108,109,110,111,112].

LAT1 is a transporter of branched-chain amino acids such as leucine, isoleucine, and valine. It is highly expressed in many cancers including breast cancers [99,113,114]. Cysteine is another metabolite that is indicative of cancer development. Elevated levels of cysteine are associated with oxidative damage and overproduction of free radicals that lead to gene mutation [115]. Dramatic metabolic shifts in choline and proline levels are known to be characteristic of metastatic breast cancer [28]. Altered levels of arginine and asparagine are also correlated with breast cancer. It has been shown that breast cancers are high L-arginine-dependent [113]. Further, it has been demonstrated that L-arginine supplementation enhances innate and adaptive immune responses and inhibits the growth of breast cancer [114]. A study in premenopausal women found an increase in plasma arginine levels to have resulted in decreased plasma levels of insulin such as growth factor 1 and estradiol [116]. Lowering the bioavailability of asparagine either by reducing dietary asparagine, or by knockdown of asparagine synthetase, reduced breast cancer metastasis [117]. These findings demonstrate that amino acid metabolism could play a vital role in cancer. Understanding how this metabolism can be utilized to prevent or treat breast cancer will be of significance. 

Some of the changes in metabolomics profile of breast cancer is presented in Table 1.

## 6. Conclusions

Metabolic reprograming is a hallmark of cancer. The field of metabolomics provides various opportunities to understand cancer initiation, promotion, progression, and metastasis. Using the metabolomics profile could result in mechanism-based prevention and treatment options for various cancers including breast cancer. Metabolic profiling is expected to differentiate between aggressive and non-aggressive breast cancers. Further, it can also predict the outcome and response to treatments. Metabolomics provides us with the knowledge of dynamic changes that occur in cancer cells, which could lead to a better understanding of the process of tumorigenesis. This could result in a major advance in elucidating whether a premalignant lesion has the potential to grow further and metastasize. This will result in reducing over-diagnosis and over-treatment. Finally, metabolomics is a highly promising field of science, which is expected to advance our knowledge of human health and reduce the burden of cancer.

## Figures and Tables

**Table 1:** Changes in metabolomics profile of breast cancer.

Study Subject	Comparison Within	Metabolomics Technique Used	Change in Metabolites (Pathways)	References
Breast cancer tissue specimen from African-American Women	Metabolites change in ER + ve versus TNBC tissue specimen	GC-MS LC-MS	Glycolysis, glycogenolysis, TCA cylcle, proliferation and redox pathways metabolites, e.g., NAD+ synthesis pathway; increase in intermediates of transmethylation were increased in TNBC compared to ER + ve.	[54]
267 Human Breast Tissue	Lipid metabolite was compared between breast cancer and normal breast tissue.	UPLC-MS/MS	Membrane phospholipids (phosphatidylcholine, phosphatidylethanolamine, and sphingomyelins ceramides) were increased in breast cancer tissue sample (more in ER-ve samples) than normal breast tissue.	[89]
Breast Cancer tissue from DUKE University Medical center	ER+ve versus ER-ve tumor	GC-MS LC-MS	Glycolytic and glycogenolytic intermediates; glutathione pathway intermediates; onco-metabolites 2-hydroxyglutrate; tryptophan metabolite Kynurenine were elevated in ER-ve tumor compared to ER +ve.	[118]
Serum sample from breast cancer patient	Change in metabolites between obese versus non-obese breast cancer patients	LC-MS	Lipid, carbohydrate, amino acid metabolism metabolites; oxidative phosphorylation, uric acid, ammonia recycling vitamin metabolism (all having role in ATP generation) are increased significantly in obese compared to non-obese breast cancer serum sample. Neurotransmitter metabolites such as serotonin, histamine; acetylcholine is also increased in obese compared to non-obese breast cancer patient serum	[119]
Plasma sample from healthy and breast cancer patient	Breast cancer patient verus healthy control	LC-MS	Increase in antioxidative metabolites (taurine and uric acid); increase in metabolites for bioenergetics (fatty acids capric acid, myristic acid); increase in three branched-chain amino acid which provides carbon for gluconeogenesis (2-hydroxy-3-methylbutiric acid, 2-hydroxy-3-methylpentanoic acid, and 3-methylglutaric acid); increase in nucleic acid biosynthesis substrate (cystidine and inosine diphosphate) in breast cancer patients plasma compared to healthy controls.	[120]
Blood (plasma) sample from healthy and breast cancer patient after overnight fasting	Plasma Metabolomics comparison carried out between breast cancer versus healthy individual	LC-MS	Arginine proline metabolism pathway metabolites and tryptophan metabolism pathway metabolites decreased and fatty acid biosynthesis pathway metabolites increases in plasma of breast cancer when compared to normal healthy individual.	[121]
Breast cancer patient tissue specimen	Comparison was made between metabolites in different sub-group of luminal A (A1, A2 and A3)	HR MAS MRS (High resolution magic angle spinning magnetic resonance spectroscopy)	Glucose signal was lower in A2 compared to A1 and A3. α-hydrogen amino acid signal was lower in A1 higher in A3 compared to A2. Al anine signal was higher in A2 compared to A3. Myo-inositol signal was lower in A1 than A2 and A3.	[122]
Breast cancer cell line MCF-7S (Adriamycin-sensitive) and MCF-7Adr (Adriamycin-resistant)	Effect of Adriamycin in metabolic profile of MCF-7S and MCF-7Adr Cell lines	GC-MS	Adriamycin significantly increases the metabolite as glucose, glutamine; amino acids such as valine isoleucine serine threonine, etc., while adriamycin slightly changed metabolites such as serine isoleucine glutamic acid after long-term exposure.	[123]
Serum sample from breast cancer patient	Comparing the metabolites in full response/pCR (pathological complete response), partial response (PR) and no response/SD (stable disease) to neoadjuvant chemotherapy	NMR LC-MS	Four metabolites were detected with threonine and glutamine decreased in pCR group compared to SD group. Isoleucine increased in pCR group compared to SD and PR and linolenic acid was decreased in pCR group and increased in both PR and SD group.	[124]
Fasting blood (serum and plasma) sample from healthy and breast cancer patients		LC-TOF-MS (Liquid chromatography time of flight mass spectrometry) GC-TOFMS (Gas chromatography time of flight mass spectrometry)	Taurine pathway metabolite (hypotaurine, pyruvate); pyruvate the metabolite for glycine, serine threonine metabolism is increased in breast cancer than in normal healthy individual. While amino acid like succinate, choline, serine, glycine and alanine and glycerol 3 phosphate, metabolite in phospholipid biosynthesis are decreased in both plasma and serum sample of breast cancer patient when compared to normal healthy individual.	[125]
